# Labial Adhesion in Postmenopausal Women: A Case Report

**DOI:** 10.7759/cureus.86300

**Published:** 2025-06-18

**Authors:** Abdul Wasay A Paracha, Vanna Giang, Andrea Shehaj, Maaz Ali, Jaime Herrera Caceres

**Affiliations:** 1 Surgery, Penn State College of Medicine, Hershey, USA; 2 Surgery, Penn State Health Milton S. Hershey Medical Center, Hershey, USA; 3 Ophthalmology, Penn State College of Medicine, Hershey, USA; 4 Ophthalmology, Penn State Health Milton S. Hershey Medical Center, Hershey, USA; 5 Emergency Medicine, Penn State College of Medicine, Hershey, USA; 6 Emergency Medicine, Penn State Health Milton S. Hershey Medical Center, Hershey, USA; 7 Urology, Penn State Health Milton S. Hershey Medical Center, Hershey, USA

**Keywords:** adhesions, labial agglutination, postmenopausal women, synechia vulvae, vulva

## Abstract

Labial adhesions are the fusion of the labia minora or majora due to an unknown etiology; however, estrogen levels and chronic infection are thought to play a role. Management can include estrogen creams, topical steroids, and surgical lysis. This case is about a 76-year-old female with a history of solitary kidney, frequent urinary tract infections (UTIs), and urethral stricture who presented with malodorous urine, dysuria, urinary frequency, abdominal discomfort, and chills. Examination showed complete fusion of the labia. UTI was ruled out, and cystoscopy, urethral dilation, and vaginoplasty were ordered. Cystoscopy discovered cystitis cystica around the trigone. A Foley catheter was placed. Follow-up recommendations included vaginal estrogen cream to treat and prevent recurrence of labial adhesions.

## Introduction

Labial adhesions, also known as synechia vulvae or labial agglutination, are the fusion of the labia minora or majora, which are located typically near the clitoris [1]. Although the underlying cause for labial adhesions remains unknown, it is believed that a state of low estrogen is a strong predisposing factor [2]. Labial adhesions occur commonly in postmenopausal women, due to low estrogen levels, leading the genital area to become susceptible to irritation and inflammation which can progress to adhesions. The risk of adhesions also increases with a patient history of diabetes mellitus, lichen sclerosis, or diminished sex. Adhesions can occur, more rarely, in reproductive age groups but is typically due to genital trauma including childbirth, sexual abuse, and genitourinary surgery [3-4].

Labial adhesions often spontaneously resolve in pediatric patients [5]; however, this is less likely in postmenopausal patients. Thus, in mild cases, management involves the application of estrogen creams or topical steroids such as beclomethasone to the labial area. If topical conservative management is ineffective, then surgical lysis of the fusion is recommended and is performed under general anesthesia at which time gentle traction is applied on the labial folds [1].

## Case presentation

A 76-year-old female with a history of having one kidney, frequent urinary tract infections (UTIs), and urethral stricture with meatal stenosis presented with symptoms suggestive of a possible UTI. She began experiencing urinary frequency and malodorous urine the previous evening, along with dysuria the following morning. Additionally, she reported chills and mild mid-suprapubic discomfort; however, she denied nausea, vomiting, chest pain, and fever. Although her urethral stricture is usually asymptomatic, episodes of infection result in the inflammation of the tissue, urinary retention, and difficulty with inserting a Foley catheter. During this visit, she had an excellent post-void residual (PVR) of 6 cc and did not exhibit signs of a UTI, prompting the decision to schedule her for cystoscopy one month later.

At the subsequent visit, cystoscopy was attempted; however, it could not be advanced into the bladder due to the narrowing of the urethral opening. In addition to this, she appeared to have labial adhesions, with her vaginal labia being completely closed and scarred. Although the small opening at the top of the labia may have corresponded with the urethra, visualization was limited due to closure of the vagina. Following a discussion with the patient regarding treatment options, expected outcomes, and associated risks and complications, the care team proceeded to schedule her for cystoscopy, urethral dilation, and vaginoplasty in one month.

**Figure 1:**
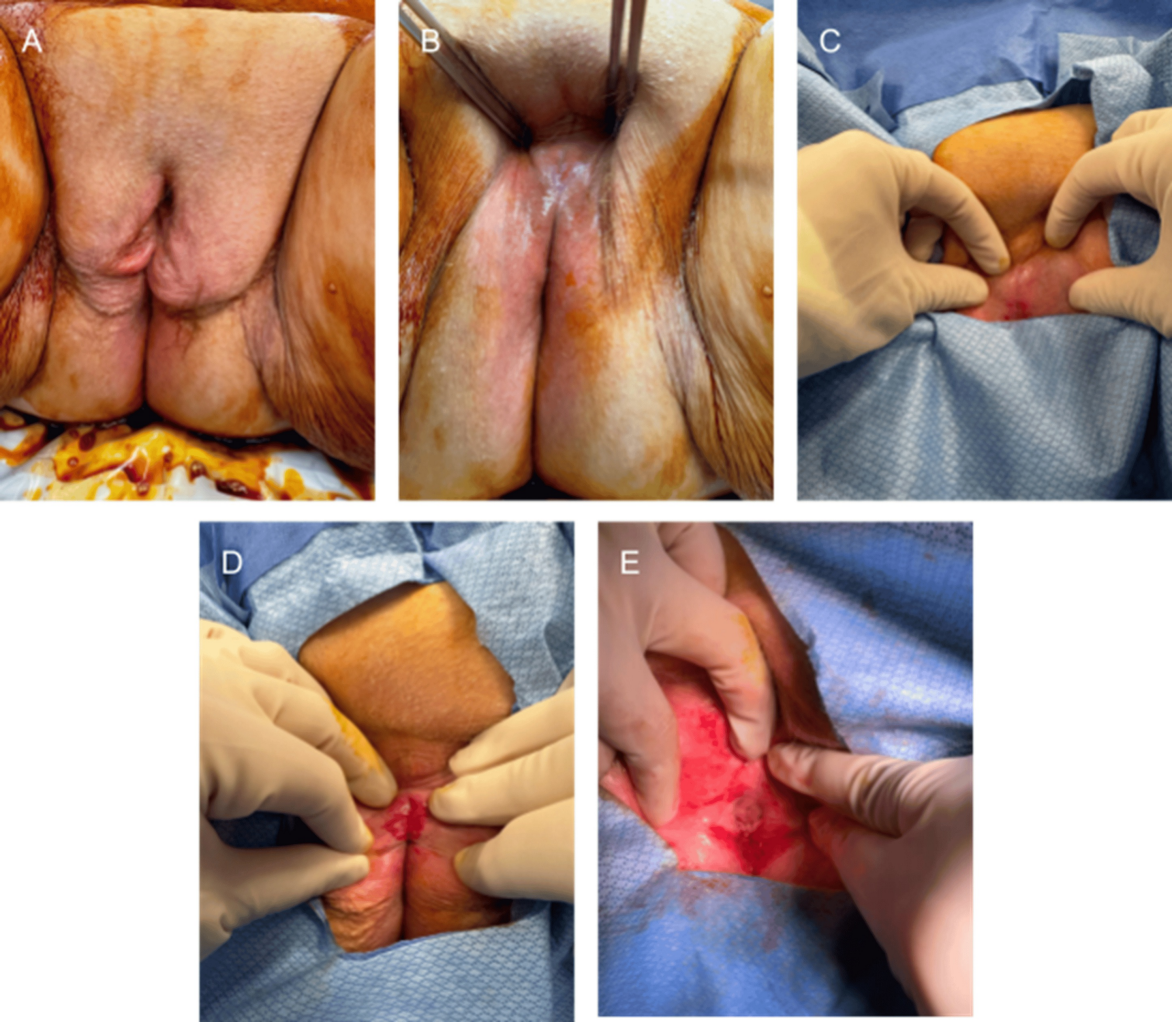
Intraoperative Progression of Labial Adhesion Management A. Vulva prior to vaginoplasty. B, C. Labial adhesions. D. Labial adhesions lysed during surgery. E. Urethral and vaginal opening after vaginoplasty.

On the day of surgery, initial examination in the operating room showed complete fusion of the labia. Using a spreading motion, the labial adhesions were lysed, allowing visualization of the urethral opening. The vaginal opening was dilated sequentially using a Foley catheter starting at 33Fr up to 43Fr. During this process, the tissue bled and appeared healthy. A 22Fr rigid cystoscope was then inserted into the urethra and bladder. No strictures or lesions, tumors, and stones were appreciated in the urethra and bladder. Signs of cystitis cystica were noted around the trigone. The surgery commenced with the placement of a Foley catheter (Figures 1A-1E). She followed up in clinic the following week with a void trial in which 240 ml of sterile water was instilled and 200 ml was voided, indicating a passed trial. At this visit, it was also recommended that she begin using a vaginal estrogen cream to treat and prevent recurrence of labial adhesions.

At her follow-up three months later, she denied urinary symptoms however mentioned experiencing significant hot flashes that began three weeks prior. Due to concerns for the vaginal estrogen causing her symptoms, she was recommended to decrease use of the cream to twice weekly. She was also referred to gynecology at this time for further evaluation. It was decided that she would continue using the vaginal estrogen cream as recommended for the time being with the understanding that another surgery could be considered if she has recurrent UTIs or persistent vaginal stenosis.

## Discussion

The exact cause of labial adhesions is not well understood; however, several factors have been proposed, including chronic vulvovaginitis, local irritation, and mechanical trauma [6]. Chronic inflammation can lead to breakdown of the vulvar epithelium, allowing the labia to fuse together. Another possible theory is believed to be related to hypoestrogenic state resulting in adhesions [7]. While low estrogen levels are a key factor, it remains unclear why labial adhesions are relatively uncommon in this age group [8]. This suggests that estrogen deficiency alone may not be sufficient, and other contributing factors such as recurrent infection, prior instrumentation, or poor genital hygiene may play a role in the pathogenesis of this disease. Although labial adhesions are reported to affect 0.6% to 1.4% of the pediatric population [9], the prevalence of this disease in the elderly population remains unknown, with few documented case reports.

Labial adhesions can present in variety of ways, depending on the extent of fusion. In most cases, a thorough physical exam is the most important diagnostic tool. Genital inspection allows clinicians to directly observe any abnormal fusion of the labia. The symptomatology of the disease can vary; however, severe adhesions can lead to vulvar discomfort, urinary incontinence, difficulty voiding, urinary retention, and dysuria [10], symptoms that were also present in this patient. A case series published in 2008 described patients with labial adhesions with most presenting with changes in their urinary stream leading to the discovery of labial adhesions [8]. Even after diagnosis, there are currently no standardized systems or guidelines to classify labial adhesions based on physical exam or symptoms, making it challenging to compare cases and therefore guide treatment decisions.

Treatment for labial adhesions typically varies from case to case depending on the severity of the symptoms. Several treatment options include topical estrogen cream, manual separation, or surgical correction. In adult patients' with severe disease, topical treatment does not always relieve symptoms, necessitating surgical correction. One study showed that surgical treatment was sufficient in five of the seven patients with severe introital infusion, with the remaining two experiencing partial recurrence [11]. As with the lack of a standard classification system for this disease process, there are also currently no treatment guidelines for labial adhesions in postmenopausal women, reflecting the rarity of this condition. Further research to help establish guidelines for both diagnosis and treatment should be performed.

## Conclusions

Labial adhesions are rare in postmenopausal women, with a thorough physical exam being the most important diagnostic tool. Management depends on the severity of symptoms. Severe adhesions may need to be treated surgically, along with continued use of topical estrogen after surgery to help with healing and reduce the risks of adhesion recurrence. This case contributes to the existing literature, aiding in the improved detection, evaluation, and management of labial adhesions in postmenopausal women.

## References

[1] Singh P, Han HC (2019). Labial adhesions in postmenopausal women: presentation and management. Int Urogynecol J.

[2] Dowlut-McElroy T, Higgins J, Williams KB, Strickland JL (2019). Treatment of prepubertal labial adhesions: a randomized controlled trial. J Pediatr Adolesc Gynecol.

[3] Norris JE, Elder CV, Dunford AM, Rampal D, Cheung C, Grover SR (2018). Spontaneous resolution of labial adhesions in pre-pubertal girls. J Paediatr Child Health.

[4] Rubinstein A, Rahman G, Risso P, Ocampo D (2018). Labial adhesions: experience in a children's hospital. Arch Argent Pediatr.

[5] O'Keefe RJ, Compton SD, Dendrinos ML, Rosen MW (2025). Management variation in pediatric labial adhesions: a retrospective cohort study. J Pediatr.

[6] Finlay HV (1965). Adhesions of the labia minora in childhood. Proc R Soc Med.

[7] Laih CY, Huang CP, Chou EC (2020). Labial adhesion in a postmenopausal female: a case report. Medicine (Baltimore).

[8] Pulvino JQ, Flynn MK, Buchsbaum GM (2008). Urinary incontinence secondary to severe labial agglutination. Int Urogynecol J Pelvic Floor Dysfunct.

[9] Norbeck JC, Ritchey MR, Bloom DA (1993). Labial fusion causing upper urinary tract obstruction. Urology.

[10] Julia J, Yacoub M, Levy G (2003). Labial fusion causing urinary incontinence in a postmenopausal female: a case report. Int Urogynecol J Pelvic Floor Dysfunct.

[11] Bradford J, Fischer G (2013). Surgical division of labial adhesions in vulvar lichen sclerosus and lichen planus. J Low Genit Tract Dis.

